# Radiation-Induced Salivary Gland Dysfunction: Mechanisms, Therapeutics and Future Directions

**DOI:** 10.3390/jcm9124095

**Published:** 2020-12-18

**Authors:** Kimberly J. Jasmer, Kristy E. Gilman, Kevin Muñoz Forti, Gary A. Weisman, Kirsten H. Limesand

**Affiliations:** 1 Christopher S. Bond Life Sciences Center, Department of Biochemistry, The University of Missouri, Columbia, MO 65211-7310, USA; kmunoz@mail.missouri.edu (K.M.F.); WeismanG@missouri.edu (G.A.W.); 2 Department of Nutritional Sciences, The University of Arizona, Tucson, AZ 85721, USA; gilmankr@email.arizona.edu (K.E.G.); limesank@arizona.edu (K.H.L.)

**Keywords:** radiation, hyposalivation, xerostomia, purinergic signaling, bystander effect, saliva, salivary gland, P2 receptors, radioprotection, head and neck cancer

## Abstract

Salivary glands sustain collateral damage following radiotherapy (RT) to treat cancers of the head and neck, leading to complications, including mucositis, xerostomia and hyposalivation. Despite salivary gland-sparing techniques and modified dosing strategies, long-term hypofunction remains a significant problem. Current therapeutic interventions provide temporary symptom relief, but do not address irreversible glandular damage. In this review, we summarize the current understanding of mechanisms involved in RT-induced hyposalivation and provide a framework for future mechanistic studies. One glaring gap in published studies investigating RT-induced mechanisms of salivary gland dysfunction concerns the effect of irradiation on adjacent non-irradiated tissue via paracrine, autocrine and direct cell–cell interactions, coined the bystander effect in other models of RT-induced damage. We hypothesize that purinergic receptor signaling involving P2 nucleotide receptors may play a key role in mediating the bystander effect. We also discuss promising new therapeutic approaches to prevent salivary gland damage due to RT.

## 1. Introduction

Advances in radiotherapy (RT) for cancer have aimed at minimizing damage to surrounding tissues through modified treatment regimens, technological improvements affording more precise RT delivery and novel radiation sources. These efforts notwithstanding, damage to surrounding tissues such as salivary glands following RT for head and neck cancer (HNC) remains a significant problem. Despite being a highly differentiated, slowly proliferating tissue, salivary glands are surprisingly sensitive to RT [1], a phenomenon attributed to disruption of the plasma membrane on secretory salivary acinar cells and apoptosis [2,3,4,5,6,7,8,9]. RT-induced salivary gland dysfunction results in hyposalivation (i.e., measured reduction in saliva production), xerostomia (i.e., the sensation of oral dryness), mucositis, nutritional deficiencies, oral infections and functional changes, such as difficulties with mastication, dysphagia (i.e., problems with swallowing) and loss of taste, which can significantly reduce the quality of life for afflicted patients [10,11]. It is estimated that >80% of HNC patients exhibit xerostomia and salivary gland hypofunction following RT [12]. Depending on the RT dose, delivery method and salivary gland-sparing techniques employed, chronic xerostomia affects 64–91% of RT patients with HNC [12,13,14]. There are limited treatment options for RT-induced hyposalivation. The muscarinic receptor agonists pilocarpine and cevimeline that induce saliva secretion from residual acinar cells [15] and artificial saliva provide only temporary symptom relief, which comes at a substantial long-term financial cost [12]. Amifostine is the only FDA-approved radioprotective therapeutic aimed at preventing damage to normal tissues, including salivary glands [16]. However, due to toxicity and potential tumor-protective effects, amifostine is not widely used [17]. Thus, development of innovative approaches to restore or retain salivary function in HNC patients receiving RT is essential [9]. The lack of treatment options to prevent dysfunction or recover function in irradiated salivary glands is compounded by a limited understanding of the underlying mechanisms and the range of variable responses to different RT regimens. In the present review, we explore the mechanisms underlying short- and long-term RT-induced salivary gland dysfunction as well as current and promising future therapeutics.

## 2. Clinical Presentation

Fractionated radiotherapy is the most common treatment regimen for head and neck squamous cell carcinoma (HNSCC), which consists of daily radiotherapy, usually 2 gray (Gy) per fraction, five days per week, to a total dose of 70 Gy to the tumor [18]. For HNC patients receiving radiotherapy, acute hyposalivation occurs within the first week after RT with a 50–60% loss of saliva flow [19]. In addition to patient-reported xerostomia, which is scored using quality of life (QoL) questionnaires, RT-induced salivary gland dysfunction is verified by an objective measure of salivary gland flow rates or scintigraphic assessment of gland function [20,21,22]. Rapid RT dose-dependent loss of secretory function seems to result from a marked loss of salivary acinar epithelial cells [23,24], a finding that has been supported in animal studies [3,4,5,6,7]. During the first three weeks after RT, nearly all patients present with mucositis due to significant inflammatory damage to the mucosal surfaces [25]. Interestingly, the incidence of mucositis is highest following fractionated radiation treatment regimens [26]. While these lesions usually resolve within a few weeks, they are reportedly painful and can be so severe as to disrupt HNC treatment regimens [11]. In addition to decreased volume, changes in saliva quality (e.g., pH, protein composition, consistency) affect the buffering capacity and digestive functions of saliva, as well as the composition of oral microbiota [27,28,29,30].

Chronic hyposalivation, usually lasting at least 6 months, is commonly experienced by HNC patients undergoing RT [12,13,14,19,31]. Chronic salivary gland dysfunction is attributed in part due to failure to regenerate functional acini and the development of glandular fibrosis [32,33,34,35,36], although the degree of acute dysfunction is predictive of long-term complications [37,38]. Chronic xerostomia is responsible for a host of complications, including functional impairments in speaking, swallowing and eating [37], poor oral clearance and altered saliva quality that lead to increased incidence of oral bacterial, yeast and fungal infections, dental caries, periodontitis [39,40], digestive disorders and nutritional deficiencies [19], which significantly reduce the quality of life of afflicted patients [20]. Thus management of chronic xerostomia is critical. Unfortunately, current management strategies are inadequate, relying on transient symptom relief afforded by artificial saliva products [41,42] or sialagogues that promote saliva production from residual acinar cells, as discussed in the therapeutics section of the present review [15]. However, there are some promising strategies for RT-induced salivary gland dysfunction, such as recently evaluated oral probiotic lozenges [30]. We will discuss novel therapies being investigated for radioprotection or salivary gland regeneration at the end of this review.

## 3. Animal Models Provide Mechanistic Insight into Radiation-Induced Salivary Gland Dysfunction

**Figure 1:**
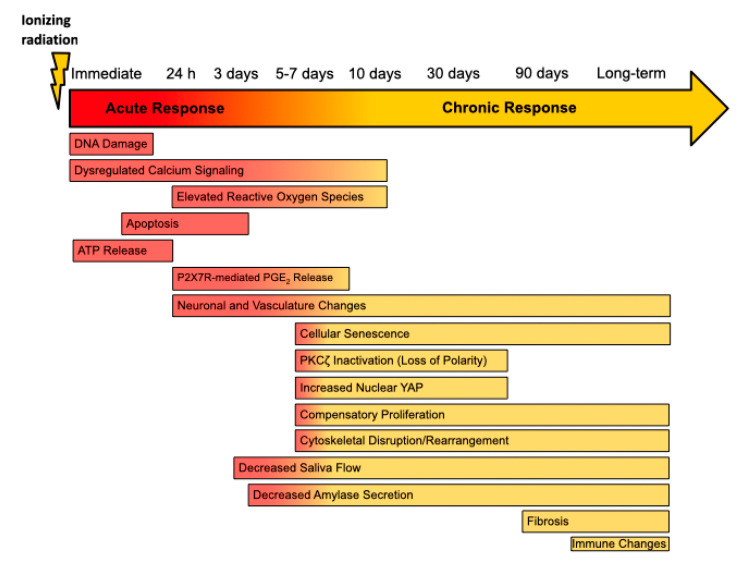
Timeline of Radiation-Induced Changes in the Rodent Salivary Gland. Following irradiation, rodent models show decreased saliva flow at approximately 3 days and a loss of amylase secretion reported as early as 4 days in rats post-IR [2,56,58]. In the acute phase, immediate DNA damage [6,59], rapid apoptosis of acinar cells [4,6,58], and elevated levels of intracellular calcium [45,46] and reactive oxygen species [45,46,60,61] contribute to acute loss of glandular function following irradiation. This period is also marked by release of ATP, which activates the P2X7 receptor (P2X7R), and P2X7R-dependent release of prostaglandin E_2_ (PGE_2_) in murine parotid cells [48]. During the transition phase, loss of apical/basolateral polarity as a result of PKCζ inactivation [55,62,63], increases nuclear Yes-associated protein (Yap) levels [55,64], compensatory proliferation [62,65,66], cellular senescence [60,67,68], and cytoskeletal rearrangements [50,69], which contribute to long-term dysfunction. Changes in innervation and vasculature have been reported as early as 24 h post-IR [53], as well as at chronic time points [54,70]. Though inconsistently reported, fibrosis generally appears between 4 and 6 months following irradiation [1,44,71]. There is little information regarding the effect of irradiation on the immune landscape of the salivary glands in rodent models, although one study indicates changes at 300 days post-IR in mice [54].

Preclinical animal models have provided many clues to the underlying mechanisms of radiation-induced salivary gland damage. Previous studies from our labs and others have utilized mouse models of ionizing radiation (IR)-induced salivary gland damage to show that acute hyposalivation detected immediately after IR and before onset of obvious gland damage is associated with aberrant calcium signaling, rapid apoptosis of acinar cells, DNA damage and enhanced reactive oxygen species (ROS) production [1,2,4,8,9,43,44,45,46,47,48]. Sustained IR-induced salivary dysfunction is additionally impacted by inflammation, neuronal and vascular changes, senescence or dysfunction of adult progenitor cell populations, cytoskeletal rearrangements and replacement of normal parenchyma with fibrotic tissue [1,44,45,49,50,51,52,53,54,55] (Figure 1). In mouse and rat models, the events giving rise to early loss of salivary gland function occur within the first 3 days following IR. Thus, we have defined acute time points for animal models as the first 3 days and chronic time points as ≥30 days post-IR. Acute IR-induced hyposalivation in mice is observed within the first few hours following IR with a marked loss of acinar cells, a decrease in saliva flow and altered saliva composition [1,4,56]. Available research investigates chronic hyposalivation anywhere from 30 to 300 days post-IR with fibrosis developing between 4 and 6 months [1,44] and as early as 30 days post-fractionated IR in minipigs [57]. Here, we summarize and discuss the signaling processes involved in IR-induced hyposalivation during both acute and chronic time points post-IR.

### 3.1. DNA Damage, Insufficient DNA Repair and Cell Cycle Arrest

Administration of IR to salivary glands activates an array of signaling pathways that influence the development of acute hyposalivation. Within minutes of IR exposure, DNA double-strand breaks were detected in mouse parotid glands using a neutral comet assay and were associated with increased phosphorylation of the H2A histone family member X (referred to as γH2AX) [59]. Furthermore, DNA strand breaks were insufficiently repaired in parotid glands due to reduced activity of the stress-induced deacetylase, sirtuin-1, that results in reduced phosphorylation of the DNA repair protein, NBS1, likely due to inadequate deacetylation that is necessary for optimum kinase function and initiation of the DNA damage response [59]. Insulin-like growth factor (IGF)-1 pretreatment in mice preserved salivary gland function following IR exposure [5]. Mice pretreated with IGF-1 had reduced γH2AX levels, increased NBS1 phosphorylation and improved DNA repair capabilities. Blocking sirtuin-1 activity using a pharmacological inhibitor in combination with IGF-1 therapy decreased DNA repair efficiency, confirming the importance of sirtuin-1-mediated DNA repair in conserving parotid gland function post-IR, especially in the context of IGF-1-mediated preservation of glandular function [59].

Following IR-induced damage, salivary glands insufficiently undergo cell cycle arrest that would allow for complete DNA repair. Following 5 Gy IR, parotid glands exhibit reduced cell cycle arrest, with a low percentage of cells in the G2/M phase at 8 h post-IR, as well as reduced levels of the cell cycle arrest gene, *p21*, at 24 h post-IR [47]. At 8 and 24 h post-IR, there were elevated levels of total and phosphorylated p53 tumor suppressor protein and increases in the truncated, inhibitory isoform of the p53 homolog, p63, (ΔNp63) which is known to block transcription of genes, including *p21*. In the IR-induced salivary gland damage model, IGF-1 pretreatment reduced salivary dysfunction in mice through induction of cell cycle arrest by increasing *p21* transcription due to reduced ΔNp63 binding and increased p53 binding to the *p21* promoter 8 h post-IR [47]. Interestingly, pretreatment of mice with roscovitine, a cell cycle inhibitor, 2 h prior to IR, increased G2/M phase cell cycle arrest and p21 protein content within 6 h post-IR [72]. Compared to vehicle treatment, roscovitine increased phosphorylation of protein kinase B (Akt), a master regulator of cell survival, and mouse double minute 2 homolog (MDM2), an E3 ubiquitin ligase that negatively regulates p53, at 6 h post-IR, which correlates with reduced apoptosis at 24 h post-IR and improved salivary output at days 3 and 30 post-IR [72]. These results confirm the importance of cell cycle inhibition immediately following IR-induced damage to enhance DNA repair and reduce apoptosis in salivary glands. 

### 3.2. Reactive Oxygen Species Generation

Reactive oxygen species (ROS) production is a known consequence of IR treatment and typically induces cellular damage immediately following IR exposure. In rats receiving 5 Gy IR, there was a significant reduction in the activity of the free radical scavenging enzymes superoxide dismutase, glutathione peroxidase and glutathione S-transferase that correlates with elevated levels of the oxidative stress markers, malondialdehyde and xanthine oxidase, as well as increased levels of peroxynitrite, nitric oxide synthase and nitric oxide in salivary glands at day 10 post-IR [61]. In mouse primary submandibular gland (SMG) cells, mitochondrial ROS levels were increased by days 1–3 post-IR with a reduction in ROS levels observed in cells deficient in transient receptor potential melastatin-related 2 (TRPM2), a calcium-permeable cation channel that is activated by oxidative stress and the DNA damage responsive protein, poly (ADP-ribose) polymerase 1 (PARP1), which correlates with improved salivary secretory function post-IR [45]. Furthermore, pharmacologically quenching ROS levels with Tempol improved salivary gland function in mice post-IR [46]. Another group showed that ROS and malondialdehyde levels remained elevated at day 7 post-5 Gy IR in SMGs, but were reduced by adenoviral induction of Sonic Hedgehog signaling at day 3 post-IR, which promoted DNA damage repair [60]. In rats receiving 18 Gy IR, there were elevated levels of the ROS-generating enzyme, NADPH oxidase at days 4–7 post-IR and increased DNA oxidation, measured as enhanced oxidized deoxyguanosine production by 4 days post-IR [58]. This phenotype was reversed following treatment with the antioxidant, α-lipoic acid, that correlated with increased amylase content and salivary function in SMGs [58]. Taken together, these results indicate that IR-induced ROS generation is detrimental to salivary gland function.

### 3.3. Dysregulated Calcium Signaling

Intracellular calcium levels are tightly regulated and impact a multitude of signaling pathways, including induction of saliva secretion, and have been shown to be dysregulated following irradiation of SMGs [45,46]. Blocking activation of the calcium-permeable cation channel, TRPM2, by pharmacologically scavenging free radicals with Tempol or inhibiting PARP1 activity, attenuates ROS production and preserves salivary gland function at days 10–30 following administration of 15 Gy IR, which was also seen in TRPM2^−/−^ mice [46]. Further evaluation of this pathway illustrated that TRPM2 activation and mitochondrial calcium uniporter (MCU) activity induced cleavage of the stromal interaction molecule 1 (STIM1) via caspase-3 activation within 48 h of IR exposure [45]. STIM1 function is necessary for regulating calcium stores in the endoplasmic reticulum and mediates store-operated calcium entry into acinar cells, with alterations in this pathway leading to reduced saliva secretion at day 30 post-IR. Blocking TRPM2, MCU or caspase-3 function with siRNA or pharmacological inhibitors reversed the hyposalivation phenotype. Likewise, adenovirus-induced expression of STIM1 at day 15 post-IR improved salivary gland function by day 30 following IR-induced damage [45]. These results suggest a key role for the regulation of intracellular calcium signaling in preserving salivary gland function post-IR.

### 3.4. Generation of Inflammatory Responses

Inflammatory responses may also contribute to IR-induced salivary gland dysfunction. Extracellular ATP (eATP), a damage-associated molecular pattern (DAMP) that commonly activates neighboring cells due to ATP release from adjacent damaged cells, is released from primary parotid gland cells immediately following 2–10 Gy IR exposure [48]. Additionally, levels of the inflammation-associated lipid, prostaglandin E_2_ (PGE_2_), are increased in parotid acinar cell culture supernatant 24–72 h following 5 Gy IR, with reduced levels of eATP and PGE_2_ release shown in mice deficient in the ATP-activated, P2X7 purinergic receptor (P2X7R), which correlates with improved saliva flow by days 3–30 post-IR [48]. Surprisingly, these pathways do not impact cell death induction in parotid glands post-IR [48], but may play a role in the inflammatory response to IR-induced salivary gland damage. It also has been observed that mRNA levels of the inflammatory cytokine interleukin (IL)-6 in irradiated salivary glands increased at 3 h post-13 Gy IR exposure and were reduced by 6 h post-IR, but increased again by day 14 post-IR. Interestingly, this increase correlated with elevated serum IL-6 levels at 6–12 h post-IR and again by day 14 [67]. IL-6 is a pro-inflammatory cytokine with diverse functions. However, the exact role that IL-6 plays in salivary glands post-IR has not been well defined. These data suggest that IR-induced damage to salivary glands leads to diverse inflammatory responses that should be further investigated.

### 3.5. Apoptosis, Autophagy and Cellular Senescence

Apoptosis of salivary acinar cells occurs at 8–72 h post-IR in mice, with the peak commonly occurring at 24 h post-IR in both parotid glands and SMGs [3,4,5,6,48,53]. Apoptosis levels have been quantitated in a multitude of ways to characterize this acute mechanistic phenotype, including via elevated mRNA expression of the apoptosis regulators Bax and Puma [4,6,73], increased caspase-3 protein cleavage [3,6,48,73,74] or enhanced caspase-3 activity [6] or via the terminal deoxynucleotidyl transferase dUTP nick end labelling (TUNEL) assay [3,48,58,73,75,76].

In rats treated by total body irradiation with 5 Gy Cesium-137, there were elevated apoptosis levels, reduced aquaporin-5 content, histological scores in SMGs indicative of tissue degeneration and a concomitant reduction in gland size and saliva secretion by days 10–30 post-IR [76]. Furthermore, in rats receiving 18 Gy IR, there were elevated levels of TUNEL-positive cells and increased cleavage of caspase-9, the upstream regulatory caspase that promotes caspase-3 activation [58]. Importantly, rats receiving α-lipoic acid treatment 1 h post-IR exhibit reduced apoptosis markers and improved saliva secretion at days 4–56 post-IR [58].

Mice lacking the tumor suppressor protein, p53, show improved salivary flow rates at days 3 and 30 following 2 or 5 Gy IR, which correlates with reduced expression of the apoptosis regulators Puma and Bax and a reduction in cleaved caspase-3 levels in histological salivary gland sections [4]. Similarly, mice with constitutive activation of Akt show reduced apoptosis of salivary acinar cells due to inhibition of p53-mediated apoptosis, which was shown to be dependent on Akt-induced phosphorylation and activation of MDM2, leading to p53 ubiquitination and degradation, reduced mRNA and protein levels of the cell cycle regulator p21 and reduced expression of the p53 homologs, p63 and p73 at 24 h post-IR [6]. Constitutive Akt activity reduced apoptosis levels 8–24 h following various doses of IR [5,6] that correlated with reduced p21 and Bax mRNA levels at 12 h post-IR, which improved salivary flow rates at days 3 and 30 post-IR [5]. Another group reiterated the importance of this pathway in mouse and human salivary gland cell cultures, with p53-mediated apoptosis being reduced in mice treated with keratinocyte growth factor-1 (KGF-1) 1 h prior to and immediately following 15 Gy IR, which correlated with improved salivary gland function and increased amylase content of saliva at 16 weeks post-IR [75].

Another study evaluated the potential use of human adipose mesenchymal stem cells (hAMSCs) to preserve salivary gland architecture and function and found that treatment of human parotid gland organoid cultures with hAMSCs increased the release of fibroblast growth factor 10 (FGF10), which reduced IR-induced (10 Gy) apoptosis measured by the TUNEL assay, decreased DNA damage as measured by γH2AX staining, and reduced levels of p53 phosphorylation, Puma and Bax protein and caspase-3 cleavage [73]. Further evaluation of FGF10 signaling showed that activation of the FGFR2-PI3K-Akt pathway increased phosphorylation of BAD and MDM2 and reduced p53-mediated apoptosis, which could be inhibited by pharmacological blockade of FGF10, FGFR2 or phosphatidylinositol-3-kinase (PI3K) activity. Injection of hAMSCs into SMGs of mice 4 weeks post-IR (15 Gy) increased amylase levels, glycoprotein content, gland weight and salivary flow rates and reduced levels of fibrosis at 12 weeks post-injection [73]. These results further support the importance of targeting p53-mediated cell death to improve salivary function post-IR.

Interestingly, knocking down expression of the apoptosis mediator, protein kinase C delta (PKCδ), in mice led to a reduction in 1 or 5 Gy IR-induced apoptosis in parotid glands 24 h post-IR [63]. Additionally, blocking the activity of PKCδ with nanoparticles containing PKCδ siRNA reduced apoptosis levels in mouse SMGs 48 h following 10 Gy IR [3]. The use of siRNA or the tyrosine kinase inhibitors, dasatinib or imatinib, to block the non-receptor tyrosine kinases c-Abl and c-Src, known upstream regulators of PKCδ, caused a similar reduction in apoptosis levels and improved saliva secretion post-IR [77,78]. Importantly, tyrosine kinase inhibition did not enhance survival or growth of HNC cell lines or tumors in mice following radiotherapy [78]. These results suggest apoptosis of salivary acinar cells is a major mechanistic component of the acute response to radiation and can occur via p53- and PKCδ-mediated apoptosis.

Autophagy is the process of “self-eating” damaged cellular components (e.g., organelles or cytoplasmic molecules) to support cell survival and healthy cell regeneration. While 5 Gy irradiation of FVB mouse salivary glands only modestly induced autophagy, pretreatment with IGF-1 followed by IR promoted autophagy activation in salivary glands as measured by conversion of microtubule-associated protein light chain 3 (LC3)-1 to LC3-II, concomitant with decreased levels of the autophagy substrate, p62, and increased interaction of the autophagy regulator Ambra-1 with Beclin-1 [56]. Notably, mice that do not exhibit autophagy in parotid acinar cells 24–48 h post-IR have increased salivary gland apoptosis levels, as well as reduced saliva flow rates that cannot be rescued with IGF-1 therapy [56]. Additionally, inhibition of autophagy leads to increased compensatory cell proliferation at 1–30 days post-IR [56]. Despite the fact that autophagosome formation was only minimally observed in irradiated salivary glands, the combined data suggest a critical role of autophagy in the damage response to irradiation, especially in the context of damage prevention using IGF-1 therapy. In a translational model utilizing miniature pigs, there is reduced levels of microtubule-associated protein light chain 3B (LC3B) and increased p62 levels in parotid glands post-IR (20 Gy) that correlates with a reduction in gland weight, acinar area, aquaporin-5 expression and saliva secretion [52]. Remarkably, activation of the Sonic Hedgehog (Shh) pathway by intraglandular delivery of adenoviral vectors expressing Shh at 4 weeks post-IR reversed this phenotype and improved saliva output in minipigs [52]. These studies suggest that further understanding of the role played by autophagy in post-IR damage may provide alternative strategies for drug development to preserve salivary gland function in HNC patients receiving RT.

Cellular senescence may play a role in IR-induced hyposalivation. Senescence has been suggested to occur in a subset of SMG cells that exhibit elevated DNA damage, measured by an increase in γH2AX+ cells and p21 mRNA by day 7 following 15 Gy IR in mice [60] and by 5 weeks following 20 Gy IR in minipigs [52]. Another study utilizing a 13 Gy dose of IR found increased levels of γH2AX+ cells, p53 binding protein-1 and mRNAs for senescence-associated markers p21, p19, decoy receptor 2, plasminogen activator-1 and IL-6 in SMGs, which were maintained above baseline 6 weeks later [67]. Interestingly, both IL-6-deficient mice and mice receiving IL-6 treatment prior to irradiation showed a reduction in these markers of senescence and improved saliva flow rates 8 weeks post-IR [67], suggesting a key role for senescence in the IR-induced damage response of salivary glands.

### 3.6. Neuronal and Vascular Changes

Alternative pathways that may be influencing IR-induced hyposalivation include damage to non-epithelial tissue within the salivary gland, such as neurons or vasculature. Importantly, parasympathetic neurons have been suggested to play a role in salivary gland regeneration post-IR damage. During embryonal development, SMGs that receive IR exposure exhibit increased epithelial and neuronal cell apoptosis at 24 and 72 h post-IR, respectively [53]. Neurturin (NRTN) is essential for parasympathetic neuronal development and survival, including in murine salivary glands [53,79,80]. Delivery of human NRTN by adenovirus serotype 5 vector (AdNRTN) to murine SMGs 24 h prior to IR (5 Gy) preserved function at 60 days post-IR [81]. Additionally, NRTN delivery by adeno-associated virus serotype 2 (AAV2) in CH3 mice and minipigs prior to IR improved saliva flow rate at 300 days and 16 weeks, respectively [54]. Treatment with NRTN has been shown to enhance parasympathetic innervation and reduce epithelial apoptosis post-IR, consistent with increased end bud formation within SMGs, supporting a potential regenerative role for neurotrophic signaling in the repair of IR-induced salivary gland damage [53]. Rats administered 18 Gy IR exhibit reduced levels of the neurotrophic factors brain-derived neurotrophic factor (BDNF) and NTRN as well as decreased levels of the neurotrophic factor receptor, GRFα2, acetylcholinesterase and neurofilament staining in SMGs, which could be reversed with α-lipoic acid treatment [70]. In minipigs following 20 Gy IR, there is a reduction in levels of BDNF, NTRN, acetylcholinesterase and the acetylcholine receptor, Chrm1, indicative of decreased parasympathetic innervation, responses that can be reversed with intraglandular adenoviral delivery of Shh at 4 weeks post-IR [52].

As for the vasculature in salivary glands, endothelial cell death occurs 4 h after 15 Gy IR, measured as an increase in caspase-3 cleavage in platelet endothelial cell adhesion molecule (CD31) positive cells, which correlates with an overall reduction in microvessel content in salivary gland tissue sections, responses modulated by treatment with the ROS scavenger Tempol for 10 min prior to IR in mice [82]. Minipigs receiving 20 Gy IR exhibit reduced blood flow and CD31 and vascular endothelial growth factor (VEGF) levels in parotid glands 20 weeks post-IR, indicative of the microvascular damage induced by IR [52]. At 90 days following 15 Gy IR, mice show an increase in blood vessel dilation in SMGs that coincides with a reduction in total capillary volume and diminished salivary function. Interestingly, co-treatment of mice with FMS-like tyrosine kinase-3 ligand (Flt-3L), stem cell factor (SCF) and granulocyte colony-stimulating factor (G-CSF) (i.e., F/S/G treatment) one month following IR promoted increased endothelial cell division, capillary content and endothelial nitric oxide synthase and endoglin expression due to bone-marrow-derived immune cell recruitment and activation of endothelial cells by F/S/G, which correlated with increased acinar cell number and saliva flow at day 90 [83]. Overall, these data suggest that repair of acute and chronic IR-induced damage to salivary glands likely requires contributions from neuronal and vascular cells.

### 3.7. Stem/Progenitor Cell Dysfunction

In addition to restoring proper innervation and vascularization, the ability of salivary glands to regain function following irradiation relies on the presence of stem and/or progenitor cells to regenerate depleted acinar cells [2]. One group reported that stem/progenitor cells are not evenly distributed within the salivary glands, but rather are localized to salivary ducts in rat and human parotid glands [84]. This suggests that preventing IR from damaging these stem/progenitor cell populations may improve salivary gland function following IR. Indeed, the same group found that irradiation of the cranial 50% of the rat parotid gland—where the authors speculate that the preponderance of progenitor cells reside—had considerably more devastating effects on saliva production at 1 year post-IR than irradiating the caudal region [84]. However, other groups have reported that progenitor cells localized to the acinar compartment of mouse parotid glands and SMGs are capable of self-renewal [55,85]. This discrepancy may be due to the markers used to identify various salivary gland progenitor cell populations. Isolation of Sca-1-, c-Kit- and Musashi-1-expressing mouse salivary gland stem cells has been achieved by in vitro culture of salispheres followed by fluorescence-activated cell sorting (FACS) enrichment using c-Kit as a marker [86]. These cells were capable of differentiating into functional amylase-producing acinar cells. This same group investigated the effect of transplanting salisphere cultures in 15 Gy irradiated female mouse salivary gland. Ninety days after salisphere transplantation, irradiated salivary glands in mice had similar morphology to non-irradiated glands and exhibited restoration of acinar cell populations and improved saliva production compared to irradiated, untreated glands [86].

Senescence as a result of IR can similarly inhibit regenerative potential. In a recent study, C57BL/6 mice receiving 15 Gy X-ray IR were treated with the senolytic drug, ABT263, by oral gavage at 8 or 11 weeks post-IR [68]. ABT263, which inhibits BCL-2 and BCL-xL, selectively eliminates senescent cells. Pilocarpine-stimulated saliva secretion demonstrated a restoration of salivary gland function in irradiated mice receiving ABT263, compared to those receiving IR and vehicle [68]. Additionally, these mice had reduced expression of senescence markers and an increase in aquaporin 5-expressing acinar cells in the SMGs [68]. The authors conclude that clearance of senescent cells promotes self-renewal of the stem/progenitor niche and restoration of salivary gland function [68]. Together, these data underscore the importance of salivary progenitor/stem cells in the regeneration of salivary gland function post-IR. These preclinical studies suggest that stem cell therapies may be a promising approach for the treatment of RT-induced hyposalivation in HNC patients.

Importantly, another group reported that regeneration following salivary gland damage due to duct ligation or under normal homeostatic conditions could occur through self-duplication of acinar cells [85]. More recently, this same group showed that while regeneration following glandular damage in mice under homeostatic conditions or following duct ligation was limited to lineage-restricted progenitors, both differentiated acinar and ductal cells in the adult mouse salivary gland were capable of contributing to acinar regeneration following irradiation [87]. Because permanent acinar cell depletion following irradiation has been reported [2], the ability of ductal cells to regenerate acinar cells is significant. If this cellular plasticity and self-duplication are also observed in human adult salivary acinar and ductal cells following radiotherapy, treatment options involving expansion or stimulation of endogenous populations without the need for isolating salivary gland stem cells may prove promising.

### 3.8. Compensatory Proliferation

Compensatory proliferation is a common reparative response to tissue damage that typically leads to replacement of dead cells following an injury. In irradiated murine salivary glands, induction of proliferation, as measured by increased expression of proliferating cell nuclear antigen (PCNA) or pKi67, is observed as early as 48 h post-IR [47,72] and continues through chronic time points (i.e., 30–90 days) [62,65,66,74]. Despite the increase in cell number, salivary glands remain non-functional post-IR, which correlates with reduced levels of salivary amylase, a marker of differentiated acinar cells, suggesting that the newly generated cells are maintained in an undifferentiated state [56,65,66]. Ectodysplasin A-1 receptor (EDAR) signaling typically occurs during embryogenesis to allow for fetal development of ectodermal tissues, such as skin, hair and exocrine glands, including salivary glands. Interestingly, activating this pathway with an EDAR-agonist monoclonal antibody restores salivary gland function post-IR, which correlates with a reduction in compensatory proliferation and increased levels of salivary amylase at days 30–90 [65]. The mammalian target of rapamycin (mTOR) is a critical signaling mediator that controls cell metabolism, growth, proliferation and survival, which is inhibited by rapamycin. Treating mice with the rapamycin analog, CCI-779, reduces proliferation rates while increasing levels of amylase and saliva flow rates at day 30 post-IR [88]. Likewise, post-IR IGF-1 treatment reduces the number of proliferating cells and enhances amylase levels and saliva secretion from days 9–90 post-IR [66].

Compensatory proliferation has been shown to be mediated by reduced activation of the apical polarity regulator, PKCζ, which leads to increased Jun kinase (JNK) signaling in parotid glands following IR [62]. The reduction in PKCζ activity is observed in a subset of stem and progenitor cells, as well as the entire acinar compartment at 5–30 days post-IR, which correlates with increased Ki67 levels [55,62]. Notably, mice lacking PKCζ have increased baseline proliferation rates that are unchanged post-IR and cannot be modulated by IGF-1 treatment [55,62]. Additionally, mice deficient in PKCζ and treated with IGF-1 do not show improvements in salivary gland function post-IR [55]. Together, these data illustrate the importance of the regulation of cell polarity and proliferation by PKCζ and its alteration due to the IR-induced damage response in parotid glands. Modulating proliferation downstream of PKCζ signaling may provide novel drug targets to preserve salivary gland function post-IR.

### 3.9. Alterations in Cell Structure

Modifications to cell junction protein interactions and actin cytoskeletal rearrangements are also observed in salivary glands following IR in mice [50] and rats [69]. Junctional regulators play a critical role in cell–cell contact and their interactions influence cell proliferation and differentiation, essential components of tissue repair. Claudins are tight junction proteins that comprise the paracellular barrier between neighboring cells and mediate intercellular permeability. In rat parotid glands, there is a transient increase in claudin-4 expression 2–3 days following 15 or 20 Gy irradiation and a reduction in levels of claudin-3 at days 7 and 30, responses that could be modulated in non-injured cells via Src kinase inhibition [69]. Epithelial (E)-cadherin is another junctional protein that is typically associated with the protein catenin, including α, β, γ or p120 isoforms that are known to play a critical role in cytoskeletal assembly and the regulation of cell adhesion, contraction and motility. A reduction in the interaction between E-cadherin and β-catenin leads to actin filament fragmentation in mouse parotid glands 7–30 days post-IR due to increased rho-associated kinase (ROCK) signaling that can be reversed by post-IR IGF-1 treatment [50]. Further evaluation of this pathway showed that ROCK signaling leads to activation and nuclear translocation of the transcriptional regulator Yes-associated protein (Yap) that is modulated by ROCK inhibition or IGF-1 treatment [64]. Yap activity is typically beneficial in injury models, although in salivary glands increased activation of Yap is seen in subsets of stem and progenitor cells, as well as the entire acinar compartment in parotid glands at days 5–30 post-IR in models that do not restore salivary function [55,64]. In contrast, post-IR IGF-1 treatment reduces Yap activity and improves salivary gland function in a PKCζ-dependent manner [55,64]. Together, these data support the mechanism whereby IR induces dissociation of tight junction proteins to promote loss of PKCζ-mediated apical/basolateral polarity, ROCK-dependent actin cytoskeletal rearrangements and loss of salivary gland function, responses that can be reversed by IGF-1 to restore salivary function of irradiated parotid glands.

### 3.10. Fibrosis

SMG biopsies from patients with advanced stage oropharyngeal cancer who received fractionated radiotherapy (1.8–2 Gy per fraction, ~35 fractions) showed glandular atrophy and periductal and parenchymal fibrosis that correlates with the degree of sialadenitis (i.e., lymphocytic infiltration of the gland). Destruction of salivary gland parenchyma with progressive replacement of functional tissue by extracellular matrix proteins impairs saliva production [33]. In rodent models, the development of fibrosis following irradiation is inconsistent, but has been reported to develop between 4 and 6 months post-IR [1,44,71]. In minipigs, IR-induced fibrosis has been reported at 30 days post-fractionated IR (200 cGy per fraction, 70 Gy total dose) [57]. Extensive fibrosis, measured by collagen deposition, was reported in CH3 mice and minipigs after 300 days and 16 weeks post-IR, respectively. In this study, CH3 mice received fractionated IR (5 × 6 Gy doses), whereas minipigs were exposed to a single 15 Gy dose of IR [54]. RNA-seq analysis of mouse SMGs 300 days post-IR revealed upregulation of genes involved in extracellular matrix remodeling and fibrosis (i.e., *Col23a1, Mmp2, Mmp3, Serping1*), whereas *Serping1* and *Mmp2* were also upregulated in minipigs at 16 weeks post-IR [54]. In a partial gland resection model utilized to investigate the mechanisms of salivary gland regeneration in the absence of confounding external stimuli, such as irradiation, genes involved in fibrotic development, ECM remodeling and the innate and adaptive immune system were similarly upregulated at days 3 and 14 post-resection in the murine SMG [89].

While humans [33,36,90] as well as mice and minipigs [1,44,54,57] show significant fibrotic damage to the salivary glands following irradiation, there is insufficient evidence to determine whether fibrosis is a cause or consequence of gland dysfunction. TGF-β, a known mediator of fibrogenesis in several tissues [91,92,93,94] is elevated in HNC patients following radiotherapy [95] and in murine models of IR-induced hyposalivation [96]. We have previously shown that TGF-β is upregulated in a mouse model of fibrosis caused by SMG excretory duct ligation and that in vivo administration of TGF-β inhibitors reduces duct ligation-induced salivary gland fibrosis [97]. TGF-β inhibition also efficiently reduces IR-induced lung [98,99] and rectal [100] fibrosis in mouse models. Further investigation is needed on the relationship of TGF-β and fibrosis to IR-induced hyposalivation and whether this pathway plays a significant role in chronic salivary gland dysfunction in RT.

### 3.11. Immunomodulation

The immunomodulatory effect of radiation on immune cells within tumors and normal tissue is a well-documented phenomenon in other models of IR-induced damage involving infiltration of immune cells of both the innate and adaptive immune system, as well as differentiation and gene expression changes within irradiated immune cell populations [101,102,103]. Lombaert et al. recently reported that female CH3 mice receiving fractionated IR (5 × 6 Gy) showed increased fibrosis, inflammation and expression of innate and adaptive immune markers (i.e., *Clec12a, Cma1, Pld4,* and *Lyz2*) in irradiated SMGs 300 days post-IR [54]. This is the first published evidence of IR-induced immunomodulation in mouse salivary glands, despite being an important area of research for other tissues and models of IR-induced damage, such as pneumonitis and pulmonary fibrosis following thoracic irradiation [102]. Similar changes in the expression patterns of these markers of immunomodulation have been shown in 15 Gy irradiated parotid glands of minipigs at 16 weeks post-IR [54].

In humans, there also is limited research on the effect of RT on salivary gland immune responses. SMG biopsies from patients receiving fractionated radiotherapy (1.8–2 Gy per fraction, 5 days per week, for a total dose of 60–70.6 Gy) revealed lymphocytic infiltration (i.e., sialadenitis), where the majority of lymphocytic infiltrates were CD3^+^ T cells with a 1:1.8 ratio of CD4^+^ to CD8^+^ T cells and significant numbers of granzyme B-stained cytotoxic T cells [33]. Macrophages and monocytes were also present, localized to the periductal and periacinar compartments.

Immunomodulation is recognized as a driver of IR-induced pneumonitis and pulmonary fibrosis [102,104]. During the acute phase, myeloid- and lymphoid-derived immune cells infiltrate lung tissue, leading to inflammation and the release of cytokines and chemokines [102]. In the chronic phase, interactions between IR-damaged tissue-resident cells, recruited immune cells and the microenvironment activate signaling pathways that promote immunomodulation, myofibroblast activation and fibrosis [102]. In IR-induced pulmonary fibrosis, CD4^+^ T cells shift from pro-inflammatory (TH1 and TH17) during the pneumonitic phase to anti-inflammatory (TH2 and T_REG_) during the fibrotic phase [102,104]. In addition to TH2 and T_REG_ cells, resident innate lymphoid cells (ILCs) are important regulators of fibrosis [105,106,107]. Notably, a unique subset of ILCs has been described in mouse SMGs [108,109], although their potential contributions to IR-induced fibrosis of the salivary gland are yet to be investigated. Nonetheless, the recent findings in CH3 mice [54], highlight the potential for future research to investigate the role of salivary gland immune cells in IR-induced hyposalivation.

## 4. Bystander Effect: Potential Role for Purinergic Signaling

**Figure 2:**
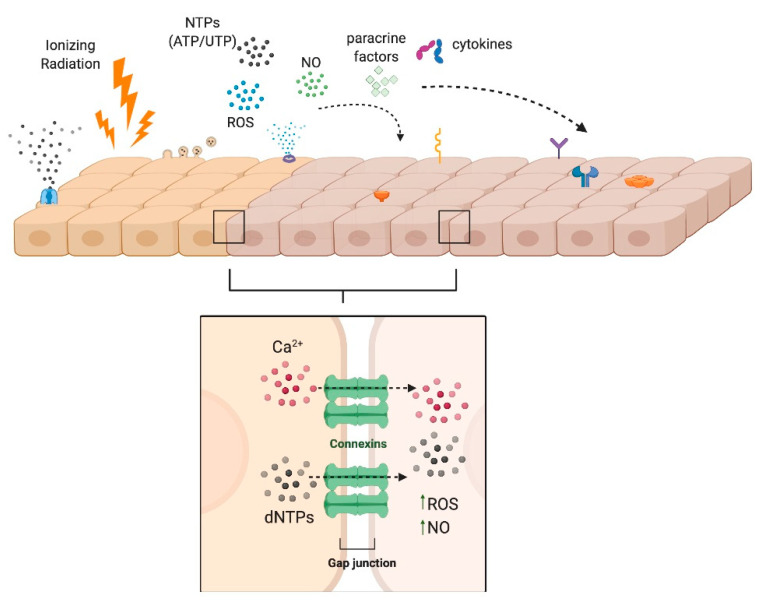
Radiation-Induced Bystander Effects. The bystander effect can be propagated by intercellular communication between adjacent cells via gap junctions or by autocrine or paracrine signaling processes whereby NTPs, nitric oxide (NO), reactive oxygen species (ROS), cytokines (e.g., TGF-β), or other second messengers elicit a response in non-irradiated cells. Created with Biorender.com.

Ionizing radiation affects cells within the radiation field and indirectly on adjacent, non-irradiated cells and tissue. This phenomenon, called the bystander effect, has been described for multiple cancer models [110,111,112,113,114] and has been investigated in relation to the protection of non-irradiated tissue [115], therapeutic approaches to cancer progression [112,116] and secondary radiation-induced neoplasms [112,117]. The bystander effect can be initiated by the release of several signaling molecules from irradiated cells, including ROS, nitric oxide (NO) or cytokines such as TGF-β1 [111,118] and through direct intercellular interactions via gap junctions or membrane channels [119,120,121] (Figure 2). While sparing techniques in cancer therapies for RT have attempted to remove salivary glands from the radiation field and/or reduce the overall radiation dose delivered to the glands, bystander effects still persist, suggesting that other radioprotective and regenerative approaches are needed [122].

**Figure 3:**
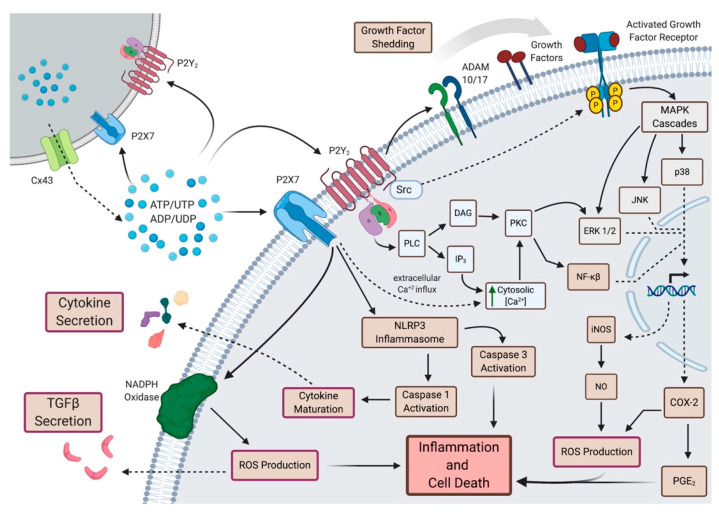
Purinergic Signaling in Bystander Effects. In response to elevated extracellular ATP (eATP) levels following irradiation, P2Y_2_R and P2X7R, which are expressed in murine and human salivary glands, may mediate a number of bystander effects. Through activation of the NLRP3 inflammasome, P2X7Rs promote ROS production, growth factor maturation (e.g., IL-1β) and subsequent release and apoptosis. Activation of either P2Y_2_R or P2X7R causes increased [Ca^2+^]_i_ and subsequent downstream signaling processes (e.g., ERK1/2 signaling, gene expression changes, inflammatory processes). P2Y_2_R activates phospholipase C (PLC) resulting in the production of inositol 1,4,5 trisphosphate (IP_3_) and diacylglycerol (DAG), in turn mobilizing intracellular Ca^2+^ and activating protein kinase C (PKC), respectively, and subsequent downstream signaling. Through its C-terminal Src-homology 3 (SH3) domain, the P2Y_2_R is involved in Src-dependent activation of growth factor receptors (e.g., EGFR, VEGFR-2) and downstream MAPK signaling as well as transactivation of EGFRs through activation of metalloproteases, ADAM10 and ADAM17. P2Y_2_Rs may also contribute to the bystander effect by promoting latent TGF-β signaling. DAG: diacylglycerol; PLC: phospholipase C; PKC: protein kinase C; ERK: extracellular signal-regulated protein kinase; JNK: c-Jun N-terminal kinase; MAPK: mitogen-activated protein kinase; COX: cyclooxygenase; PGE_2_: prostaglandin E_2_; ADAM: A Disintegrin And Metalloprotease. Created with Biorender.com.

In the past ten years, mounting evidence demonstrates that purinergic signaling can mediate the IR-induced bystander effect in adjacent, non-irradiated cells [110,122,123,124,125,126,127,128]. Extracellular nucleotides such as ATP (eATP), which are released into the extracellular space in response to cellular damage including ionizing radiation [124,128], act as autocrine or paracrine signaling molecules via activation of P2 receptors (P2Rs) on nearby cells [122,123,124,128,129,130,131,132,133,134]. In response to γ-irradiation, both human and murine cell lines have been shown to release ATP, thereby activating G protein-coupled P2Y [110,129,135] and ATP-gated ionotropic P2X receptors [126,136], which initiate purinergic signaling. Although no studies have yet investigated this bystander effect in IR-induced salivary gland dysfunction, we and others have reported on the expression of P2Y_2_, P2X7 and P2X4 receptors in salivary gland epithelia of mice [137,138,139] and humans [139], suggesting that irradiated salivary glands in vivo should be highly sensitive to elevated levels of IR-induced eATP release. Mechanisms of P2 receptor signaling relevant to the bystander effect have been investigated in multiple tissues [110,122,123,124,125,126,127,128] and their potential roles in IR-induced salivary gland damage are summarized in Figure 3.

### 4.1. ATP Release

In addition to responding to elevated eATP, the P2X7 receptor (P2X7R) has been implicated in the IR-induced release of ATP. As previously described in B16 murine melanoma cells [124], we recently demonstrated that γ-irradiation of parotid epithelial cells induces release of eATP in a P2X7R-dependent manner [48]. One way that IR induces the release of eATP is through upregulation of connexin 43 (Cx43) [140], a gap junction hemichannel involved in intercellular communication [141], as well as eATP release [142,143]. The activation of P2X7R by eATP induces an increase in the cytoplasmic calcium concentration, [Ca^2+^]_i_, which promotes eATP release via Cx43 [128]. Indeed, Cx43-mediated release of ATP following irradiation of B16 melanoma cells is dependent on P2X7R [123]. As reported, IR-induced ATP release through Cx43 could be suppressed by blockade of P2X7R or downstream purinergic signaling pathways, including tyrosine kinase and Rho kinase activation, actin cytoskeletal rearrangements and increases in [Ca^2+^]_i_ and ROS production [123]. Together, these data demonstrate a role for P2X7R in mediating release of eATP following IR.

### 4.2. Dysregulated Calcium Signaling

Once released, eATP can then bind available P2 receptors, such as P2X7Rs or P2Y_2_Rs, and promote a host of signaling processes. As we discussed earlier, a rapid increase in [Ca^2+^]_i_ is observed following irradiation of submandibular glands in mice via store-operated Ca^2+^ entry (SOCE), a response that was abrogated in TRMP2^-/-^ mice [45]. Activation of either P2X7R or P2Y_2_R by eATP leads to elevated [Ca^2+^]_i_ though by different mechanisms. Like TRPM2, P2Y_2_Rs promote cytoplasmic entry of Ca^2+^ via SOCE [144], while P2X7Rs mediate extracellular Ca^2+^ influx [138]. Calcium signaling contributes to many of the signaling processes underlying bystander responses [145,146,147] that we will discuss in more detail below, including MAPK signaling [148], ROS production [146], NF-κB-mediated iNOS synthesis [149] and COX-2/PGE_2_ signaling [149,150,151,152]. A rapid increase in [Ca^2+^]_i_ was observed in bystander cells that were exposed to conditioned medium (CM) from irradiated human keratinocytes [146], glioma and fibroblasts [148]. In human keratinocytes, the apoptosis observed in bystander cells following exposure to CM was blocked by treatment with the calcium chelator, EGTA, suggesting influx, not SOCE, was important for the observed bystander effect [146]. Because of the central role of Ca^2+^ signaling in mediating bystander effects, the contributions of P2 receptors to IR-induced increases in [Ca^2+^]_i_ following irradiation of the salivary gland should be further investigated.

### 4.3. MAPK Signaling

A number of groups have implicated MAPK signaling in propagating bystander effects in non-irradiated cells [146,147,153]. Using human B-lymphoblastoid cell lines, one group reported phosphorylation of ERK1/2, JNK and p38 in bystander cells exposed to CM from X-ray irradiated cells [153]. Additionally, bystander-induced caspase 3/7 activation could be diminished by treatment with ERK inhibitor U0126, JNK inhibitor SP600125 or p38 inhibitor SB203580. Human keratinocytes treated with conditioned medium (CM) from irradiated keratinocytes showed elevated ERK and JNK signaling [146]. CM-mediated apoptosis in bystander keratinocytes could be blocked by JNK inhibition, although ERK inhibition seemed to increase bystander-induced apoptosis in these cells [146].

G_q_-coupled P2Y receptors (P2Y_1,2,4,6,11_Rs) activate phospholipase C (PLC) resulting in the production of inositol 1,4,5-triphosphate (IP3) and diacylglycerol (DAG), in turn mobilizing intracellular Ca^2+^ and activating protein kinase C (PKC) and subsequent downstream signaling pathways [144], including ERK1/2 activation, that have been implicated in mediating the bystander effect [146,154]. Further, through its C-terminal Src homology 3 (SH3) domain, the P2Y_2_R is involved in Src-dependent activation of growth factor receptors (e.g., EGFR, VEGFR-2), ERK1/2, and JNK [144]. P2Y_2_R is also capable of transactivation of EGFRs by activating metalloproteases (i.e., ADAM10 and ADAM17) [155]. In turn, EGFR activation initiates MAPK signaling cascades. In this way, P2Y_2_Rs are capable of mediating MAPK signaling through canonical G_q_-coupled signaling or through activation of growth factor receptors such as EGFR.

Another potential role for P2Rs in mediating bystander effects involves PKC. We discussed above that knocking down PKCδ expression inhibits IR-induced apoptosis in irradiated murine parotid glands [63], while blocking PKCδ activity by administration of nanoparticles containing siRNA targeting PKCδ reduces apoptosis in irradiated mouse SMG [3]. Directly blocking c-Src with imatinib or dasatinib had a similar anti-apoptotic effect and enhanced saliva production [78]. P2R antagonists (DIDS, suramin and Cibacron Blue 3GA) increase phosphorylation of PKCδ in rat parotid acinar cells, confirming that P2 activity promotes PKCδ activation [156]. Together these data suggest that ATP-induced activation of P2Rs promotes apoptosis in bystander cells via PKCδ phosphorylation.

### 4.4. COX-2/Prostaglandin E_2_ Signaling

The inflammatory cyclooxygenase-2 (COX-2) signaling pathway that is required for PGE_2_ synthesis has been implicated in promoting bystander effects [149,150,151,152,157]. Most bystander studies involve the transfer of conditioned medium from irradiated cells to non-irradiated cells in vitro. Zhou et al. used a novel mylar strip culture dish to shield a portion of human fibroblasts from irradiation while remaining in the same culture vessel as irradiated cells [150]. Using this technique, bystander human fibroblasts showed overexpression of COX-2 [150]. Inhibition of COX-2 with NS-398 decreased the bystander effect in non-irradiated cells, as determined by the frequency of mutations in the hypoxanthine-guanine phosphoribosyltransferase (HPRT^-^) locus and the percent of surviving cells [150]. They also demonstrated that inhibition of ERK1/2, which lies upstream of COX-2, could diminish the bystander effects. More recently, this same group reported that NF-κB was important for mediating bystander effects in human fibroblasts, which could be attributed to NF-κB-mediated inducible NO synthase (iNOS) and COX-2 expression [149]. The NF-κB inhibitor, Bay 11-7082, and 2-(4-carboxyphenyl)-4,4,5,5-tetramethylimidazoline-1-oxyl-3-oxide, a scavenger of NO, decreased mutation frequency in bystander cells [149].

PGE_2_ has also been suggested to mediate the bystander effect [147,157]. We have previously reported that PGE_2_ levels are increased in parotid acinar cell culture supernatant 24–72 h post-IR [48]. We also reported reduced levels of PGE_2_ release in mice lacking P2X7R [48]. In addition to these findings, P2X7Rs and P2Y_2_Rs lie upstream of many of the signaling pathways resulting in expression of COX-2 (Figure 3). Together, these findings suggest that COX-2/PGE_2_-mediated bystander effects are dependent on signaling through P2X7Rs or P2Y_2_Rs.

### 4.5. DNA Damage Response

Elevated levels of γH2AX are an indicator of DNA damage, a well-documented response to ionizing radiation [59]. In irradiated A549 human lung cancer cells, elevated γH2AX levels were abrogated by pretreatment with the ectonucleotidase apyrase [158], a response that could be eliminated by the ectonucleotidase inhibitor ARL67156 or addition of ATP or UTP. Similar to our findings in primary murine parotid epithelial cells [48], ATP is released from A549 cells following γ-irradiation (2 Gy) in a P2X7R-dependent manner. The finding that antagonism of P2Y_6_R, P2Y_12_R or P2X7R blocked activation of DNA repair mechanisms led to the overall hypothesis that P2X7R-dependent release of ATP and subsequent autocrine activation of G protein-coupled P2Y receptors was responsible for the observed DNA damage response [158]. Additionally, downstream P2R signaling molecules, such as nitric oxide (NO), are important mediators of radiation-induced DNA damage in bystander cells [128,159].

### 4.6. Reactive Oxygen Species and TGF-β1

ROS and TGF-β1 are well-appreciated mediators of the bystander effect in multiple IR models [160], serving as second messengers in an autocrine or paracrine fashion. In response to ATP, P2X7Rs regulate production of ROS by NLRP3 inflammasome activation [161] and ROS promote TGF-β1 release [162]. P2Y_2_Rs may also contribute to the bystander effect by promoting latent TGF-β signaling. TGF-β, latency activated protein (LAP) and latent TGF-β binding proteins (LTBP) form a covalently-bound large latent complex (LLC) that is secreted from cells, whereupon it binds to integrins (e.g., α_v_β_5_, α_v_β_6_, α_v_β_3_ and α_v_β_8_) in the extracellular matrix (ECM) through LAP interaction with RGD sequences. Latent activation of TGF-β signaling can occur through metalloprotease-dependent cleavage of LAP or LTBP or disruption of LAP-RGD interaction with integrins [163,164,165,166]. P2Y_2_R activation also induces metalloprotease activity [167,168] and the P2Y_2_R contains an extracellularly-oriented RGD sequence that enables its direct binding and activation of the α_v_β_5_ integrin [167]. Through these mechanisms, activation of P2Y_2_R or P2X7R could promote the TGF-β1 and ROS-mediated bystander effects.

### 4.7. Immunomodulation

There is currently insufficient research regarding the role of immunomodulation in IR-induced salivary gland dysfunction or in models of IR-induced bystander effects to fully define the role of P2 receptors. A T cell-mediated mechanism of long-distance effects on metastatic lesions following irradiation of primary tumors—coined the abscopal effect—has been described [169,170,171]. Most bystander effect studies are performed in vitro, making it difficult to ascertain the contributions of various immune cells that recapitulate in vivo conditions [172]. However, one study utilized an in vivo model of radiation-induced acute myeloid leukemia to investigate the bystander effect. They found that the long-term bystander consequences of irradiation in vivo were due to altered macrophage activity [172]. P2Y_2_R signaling pathways are involved in the recruitment, migration and proliferation of immune cells [173,174,175], and thus activation of this receptor by elevated eATP levels following IR should mediate the modification of the immune landscape in the salivary gland.

In conclusion, the available data strongly support the notion that purinergic signaling plays a role in the IR-induced bystander effect in multiple models. In particular, the role of P2X7R, P2X4R, and P2Y_2_R, given their reported expression in human and mouse salivary glands, should be further investigated for their potential as novel targets for the prevention of the bystander effects underlying IR-induced salivary hypofunction.

## 5. Therapeutics

As mentioned above, treatments for RT-induced hyposalivation are limited to sialagogues, such as pilocarpine and cevimeline, which induce saliva secretion from residual acinar cells, artificial saliva and the single FDA-approved radioprotective therapeutic, amifostine. There are also a number of salivary gland radiation sparing techniques that have been utilized to minimize IR-induced damage. Promising new radioprotective and regenerative approaches are being investigated in preclinical animal models. The next section summarizes current and promising therapeutic approaches to IR-induced salivary gland dysfunction.

### 5.1. Salivary Gland-Sparing Techniques

#### 5.1.1. Dosing Strategies

Fractionated radiation is the primary method of radiotherapy for cancer patients, including for the treatment of HNC, but causes cell toxicity and other severe side effects. Fractionated radiation divides the total curative dose into a series of smaller doses, which allows the patient to better tolerate and maintain the treatment. Conventional fractionated radiotherapy for HNC patients is commonly prescribed as 2 Gy per day, five days per week, for multiple weeks, to a total dose of 70 Gy [18]. Multiple small radiation doses allow non-tumor cells to recover between IR exposures, while more severely damaging malignant cells due to their high proliferation rates. A meta-analysis, including 34 trials and 11,969 HNC patients (with primarily late-stage tumors of the oropharynx and larynx), comparing different subtypes of fractionated radiation found that hyper-fractionated radiation, where a patient receives the same total curative dose of radiation, but in two fractions per day (totaling 4 Gy/day), showed improved overall survival and progression-free survival when compared to conventional fractionation (2 Gy/day). However, these more intensive radiation schedules induced more severe side effects, including increased instances of mucositis, which caused RT patients to go on feeding tubes more frequently [176]. Overall, hyper-fractionated radiation was less feasible than conventional approaches due to the cost, scheduling difficulties and the increased severity of side effects. Other altered fractionation dosing schedules, including increasing the IR doses per day and shortening the total time to curative dose, were evaluated, but these treatments did not show improvement in disease outcomes or sparing organs at risk compared to conventional fractionation [176]. While fractionated therapies are more efficacious as cancer treatments, there is currently no evidence that hyper-fractionated radiotherapy or other altered fractionated dosing schedules have any effect on decreasing the incidence of xerostomia [19,176].

#### 5.1.2. Intensity Modulated Radiation Therapy (IMRT)

IMRT is a form of radiotherapy for tumors with advanced precision and dosing control. IMRT utilizes three-dimensional (3D) imaging of tumors, typically by computerized tomography (CT) or magnetic resonance imaging (MRI), to design beam patterns with varying intensities to direct at the tumor with the goal of sparing non-malignant tissues, especially radiosensitive tissues, such as the brain, spinal cord and salivary glands [177]. These complex, variable RT patterns aim to keep the total dose to below 26 Gy for parotid glands [178,179] and 39 Gy for submandibular glands [180] to spare gland function without decreasing the dose to the tumor. Computer-calculated dosing and beam angles are defined with the 3D images and the beam is typically targeted at the tumor site with image guidance from CT or X-ray scans of the patient to deliver varying beam doses across the tumor in a fixed field. IMRT controls tumor growth better than or similarly to 3D-conformal radiotherapy (CRT). A study looking at tumor recurrence in 3D-CRT- versus IMRT-treated HNC patients found that those receiving IMRT following surgical resection of the primary tumor had improved tumor control two years post-treatment compared to patients receiving 3D-CRT; however, treatment modalities showed no difference in tumor recurrence following definitive radiotherapy [181]. Conversely, a meta-analysis of studies evaluating disease-free survival and overall survival in patients receiving 3D-CRT or IMRT found that there was no difference in outcomes [182]. Compared to conventional radiotherapy, IMRT is substantially more time-intensive, with the need for extensive planning and increased clinician and machine time [177]. However, IMRT reduces non-malignant tissue radiation exposure and improves the QoL for HNC survivors post-therapy [182]. The first study to evaluate differences in xerostomia rankings across patients receiving 3D-CRT versus IMRT found that patients who underwent IMRT, while still experiencing xerostomia, had significantly improved scores at all times during and following radiotherapy [183]. Despite the improvements in sparing salivary glands, IMRT still leads to hyposalivation and xerostomia and has been shown to alter saliva composition, including pH and electrolyte content; however, these patients have improved saliva output one year after ending treatment [184].

#### 5.1.3. Volumetric Modulated Arc Therapy (VMAT)

VMAT, also known as Rapid Arc therapy, is a recently developed form of radiotherapy that continuously exposes the tumor to a radiation beam while rotating in an arc shape around the tumor. VMAT improves radiation targeting by more precisely controlling rotational speed, shape and dose rate, where radiation beams can be directed as a single or double beam towards the tumor site and can rotate in a full or half arc [185]. One major benefit of VMAT is the efficiency in delivering radiotherapy and the reduction in machine time, when compared to IMRT; however, extensive planning for targeting radiation arcs to the tumor is a limitation [185]. Radiation dosing measurements between VMAT and IMRT were compared and IMRT offered greater homogeneity while VMAT had increased conformity, although both therapies used similar dosing strategies for planning target volume and VMAT showed reduced radiation exposure to organs at risk, including salivary glands, brain stem, spinal cord and the oral cavity [186]. These results were supported by a more recent study that slightly favored VMAT, particularly in the context of increasing the patient’s QoL [187]. Although VMAT is a relatively recent development in radiotherapy [185], there have been studies over the last decade offering improvements in VMAT, such as auto-planning to precisely target tumors while conserving organs at risk. Manual contouring around organs at risk is a standard planning objective of VMAT to avoid radiation exposure to non-malignant regions, but this is time and labor intensive. Auto-contouring, accomplished with a computer system, has been combined with simplified contouring, which uses simple drawn structures of organs at risk to develop VMAT plans with acceptable dosing strategies for organs, specifically salivary glands, that can be completed in a more efficient time as compared to manual contouring (2 min for auto-planning VMAT versus 7 min for manual) [188]. In auto-planning VMAT, computer calculated dosing strategies are generated with input on planning target volume as well as contours of organs at risk that require minimal radiation exposure. Computational plans can then be manually edited by clinicians to better define therapy components. Auto-planning VMAT shows similar or improved dosing characteristics for tumors, reduces exposure to organs at risk and requires less clinician time, when compared to manual planning [189]. These studies show the continuous improvements being made in radiotherapy techniques, with VMAT becoming a more accessible and efficient treatment option for HNC patients, especially in areas with limited medical resources.

#### 5.1.4. Proton Beam Radiotherapy (PBRT)

PBRT is a relatively new and alternative form of radiotherapy that focuses protons in a beam to target a tumor site. Compared to photons, protons have unique physical characteristics and decelerate very quickly as they travel through matter, exhibiting a phenomenon referred to as the Bragg peak [190]. This difference allows for more precise targeting of protons to the malignancy and reduces potential damage to organs at risk, such as salivary glands. In a recent study comparing the development of secondary tumors following 3D-CRT, IMRT or PBRT, 3D-CRT and IMRT had similar rates of cancer recurrence, whereas PBRT showed reduced recurrence across cancer types [191]. Unfortunately, proton therapy is currently expensive and understudied, making it a less ideal option for most clinicians and patients [192]. More research evaluating the differences in tumor control and damage to organs at risk with PBRT versus IMRT or VMAT should be conducted to further validate the efficacy of this alternative type of radiotherapy.

#### 5.1.5. Intraoral Stents and Temporary Submandibular Gland (SMG) Transplantation

Two additional non-pharmacological salivary gland-sparing interventions have been utilized, i.e., intraoral stents and temporary submandibular gland transplantation. Intraoral stents are personalized medical devices designed to position and stabilize the oral cavity to prevent unnecessary irradiation of adjacent tissues. These devices are easy to manufacture [193] and have been demonstrated to efficiently reduce irradiation of off-target tissues [194,195,196]. Salivary glands can be protected from irradiation by temporarily relocating them further away from the field of irradiation in a procedure known as temporary SMG transplantation. This surgical alternative has been shown to be safe and cost effective and involves releasing the SMG from surrounding tissues and temporarily repositioning it in the submental space over the digastric muscle, thereby removing it from the radiation field [197]. Pathak et al. showed that in patients receiving RT, those that underwent temporary SMG transplantation had no significant differences in salivary flow rates before RT compared to those who did not undergo transplantation [168]. After RT, 73% of the group that received the transplantation had a preserved mean salivary flow rate, compared to 27% of the group that did not receive transplantation [198]. Despite its success, due to the risks of co-transplantation of malignant tissues and subsequent relapse or secondary metastases, SMG transplantation is only used in RT for specific types of head and neck cancers.

Recently, novel salivary glands have been identified in humans, dubbed the tubarial glands due to their proximity to the torus tubarius [199], in a retrospective analysis of PET/CT scans of 100 prostate or para-urethral gland cancer patients using radiolabeled ligands that bind prostate-specific membrane antigen (PSMA), which is highly expressed in all major and minor salivary glands [199,200]. The tubarial glands were described as predominantly mucous gland tissue with multiple draining ducts located in the dorsolateral pharyngeal wall. The sparing techniques we describe in this review have not taken into consideration these glands and none of the current targeted techniques avoid these structures that lie posterior to the nasopharynx [199]. The effect of RT dose on tubarial glands was further investigated with the incidence of xerostomia and dysphagia found to be correlated in 723 HNC patients at both 12- and 24-months after initial physician-rated xerostomia. This exciting discovery raises the question of whether modifying radiation fields to spare the tubarial glands will prevent RT-induced xerostomia. As of yet, these glands have not been identified in any preclinical animal models and it is noteworthy that mice lack the torus tubarius [201]. However, if similar glands are present in animal models, we anticipate that future studies will examine possible RT field modifications to optimize radioprotective therapies that preserve glandular function.

Radiation treatment plans for HNC are not one size fits all and depend on many factors, such as cancer type, location, stage and the patient’s overall health. Comparisons of multiple types of radiotherapy plans for HNC have been performed and researchers found that there are benefits to certain planning methods, depending on the type and stage of HNC [202]. Furthermore, while there have been substantial improvements in RT techniques in recent years to reduce exposure to organs at risk, patients still exhibit side effects, such as hyposalivation and xerostomia, which can lead to multiple complications, as discussed earlier [1]. Further evaluation of mechanisms of radiation damage to salivary glands to develop novel radioprotective and/or regenerative approaches is, therefore, necessary to improve the quality of life for HNC survivors.

### 5.2. Symptom Relief

#### 5.2.1. Artificial Saliva

Radiation-induced xerostomia is influenced by factors including the patient’s salivary gland health and function prior to treatment, the magnitude of the treatment and the individual response of the patient. Current strategies to manage RT-induced xerostomia provide only short-term relief. Artificial saliva products have played a limited role in the treatment of xerostomia due to their extremely transient nature. Despite human saliva consisting of approximately 99.5% water, the proteins, lipids, ions and other biomolecules that compose the remaining 0.5% are essential and have yet to be efficiently mimicked artificially [41]. Spirk et al. conducted a small clinical study evaluating the three most utilized artificial saliva products, characterizing their physiochemical properties in comparison to unstimulated human saliva [42]. Their study demonstrated that these artificial saliva products differed significantly from human saliva in pH, osmolarity and/or electrical conductivity [42]. Their findings explain why the utility of artificial saliva or saliva substitutes is limited.

#### 5.2.2. Sialagogues

Treatments to stimulate the function of remaining salivary gland tissue have had more success than artificial saliva. Systemic sialagogues stimulate saliva secretion from residual, functional salivary gland acinar cells to compensate somewhat for decreases in saliva flow due to RT and, thus, their effectiveness depends heavily on the number of surviving acinar cells [203]. Both pilocarpine and bethanechol are systemic sialagogues that activate muscarinic-cholinergic receptors. A phase III randomized clinical trial studying the effects of pilocarpine therapy after RT in HNC patients demonstrated that unstimulated saliva flow in the patients who received pilocarpine therapy was significantly higher than in those receiving a placebo [204]. The authors reported that this increase in saliva flow did not correlate with improved QoL scores and also concluded that pilocarpine therapy had no significant effect on the incidence of mucositis. These observations support previous clinical studies [205,206,207]. Cevimeline, a quinuclidine derivative of acetylcholine that selectively activates M3 muscarinic receptors, has obtained FDA approval for xerostomia treatment in Sjögren’s syndrome patients, although its use for RT-induced xerostomia remains off-label [21,208]. In a prospective, single-arm study with 255 participants, cevimeline taken orally for 52 weeks improved symptoms in 59.2% of HNC patients who received RT [209]. The benefit of cevimeline over pilocarpine is that its half-life is much longer [210], offering prolonged benefit to patients. Similarly, bethanechol is a stable analogue of acetylcholine and, thus, its effects last longer, as it undergoes slower degradation by cholinesterases. Similar to pilocarpine, bethanechol stimulates the parasympathetic nervous system by activating muscarinic receptors. Although still only indicated for post-operative or postnatal urinary retention, studies by Epstein et al. and Gorsky et al. demonstrate that post-RT bethanechol therapy is just as effective as pilocarpine therapy [211,212]. Patients receiving either bethanechol or pilocarpine reported a 39% or 33% subjective increase in unstimulated saliva production, respectively [212]. Patients receiving a combination therapy of bethanechol and pilocarpine did not show statistically significant improvements over either monotherapy. The authors postulate that this is due to “parenchymal fatigue”, hinting at a saturation limit of the remaining healthy tissues [212]. Jham et al. was one of the first studies to evaluate bethanechol therapy concomitant with receiving RT in regards to preventing rather than ameliorating xerostomia [213]. They observed that the use of bethanechol throughout the duration of RT led to a statistically significant increase in whole resting saliva secretion at the culmination of RT, compared to a control group receiving artificial saliva. Similarly, a double-blind randomized clinical study [214] evaluating the utility of bethanechol therapy concomitantly with or after RT demonstrated that patients receiving bethanechol reported improved xerostomia symptoms and had statistically significant increases in unstimulated and stimulated whole saliva flow [214]. Despite many promising studies with a diverse cohort of HNC patients, there is still a lack of longitudinal studies. A systemic meta-analysis assessing the effect of pharmacological interventions for RT-induced xerostomia found that there was insufficient data to support any effect of pilocarpine therapy on salivary gland function or QoL [215]. The authors noted that what data were available were of very low quality, supporting the need for additional studies. As the time of writing this review, there have been no clinical studies evaluating treatment outcomes utilizing muscarinic receptor agonists beyond a few months post-RT. Given the lifelong presentation of post-RT xerostomia, therapies need to be effective over an extended period of time. Additionally, because of the transient effect of artificial saliva and sialagogues, these treatment options present a significant financial burden [12].

### 5.3. Radioprotection

#### 5.3.1. Amifostine

**Figure 4:**
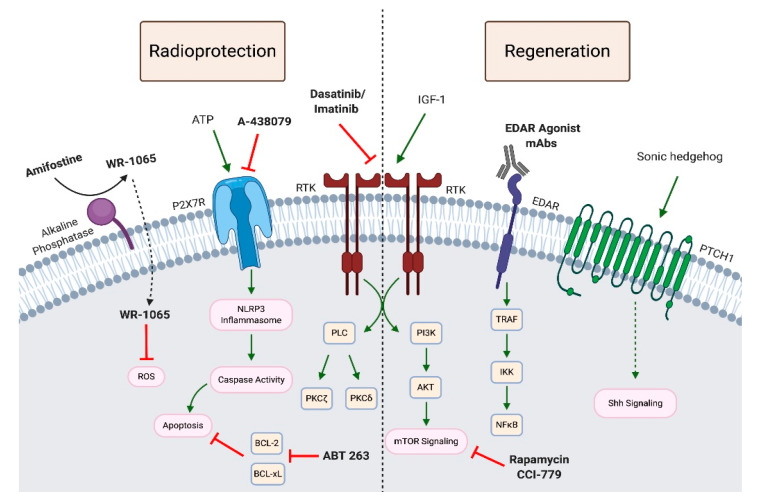
Pharmacological Approaches to Salivary Gland Radioprotection and Regeneration. Amifostine is currently the only radioprotective therapeutic approved for the prevention of RT-induced hyposalivation. The membrane-bound alkaline phosphatase converts Amifostine to WR-1065 that is then taken up by the cell. WR-1065 is thought to promote radioprotection by scavenging reactive species in turn affecting gene expression, apoptosis, chromatin stability, DNA damage repair and enzymatic activity [221,222]. Other promising radioprotective therapeutics being investigated in preclinical animal models include the P2X7R antagonist, A-438079 [48], and the tyrosine kinase inhibitors, dasatinib and imatinib [77,78]. Pharmacological approaches to regeneration studied in animal models post-IR target a number of signaling pathways. IGF-1 treatment 4–7 days post-IR restored saliva production in a PKCζ-dependent manner [55]. mTOR signaling is another target that has been investigated by several groups to promote salivary gland regeneration [88,223]. Administration of the rapamycin analog, CCI-779, following IR improved saliva flow rates at 30 days post-IR [88]. Transient upregulation of Shh signaling by either overexpressing a Shh transgene or by administering a smoothened agonist, restored stimulated saliva flow [224]. EDAR agonists, such as monoclonal antibodies that promote EDAR signaling, are essential for salivary gland development and have shown promise in restoring salivary gland function in mice [65]. The senolytic agent, ABT263, which depletes senescent cells by inhibiting BCL-2 and BCL-xL, has been shown to promote salivary gland regeneration and self-renewal capabilities of residual salivary gland stem cells [68]. RTK: receptor tyrosine kinase; PLC: phospholipase C; PKC: protein kinase C; BCL: B-cell lymphoma; TRAF: tumor necrosis factor receptor-associated factor; IKK: IkB kinase; AKT: protein kinase B; EDAR: ectodysplasin A-1 receptor; PTCH: patched receptor. Created with Biorender.com.

While drugs such as pilocarpine and cevimeline have been approved by the FDA to treat xerostomia, amifostine was the first and currently the only FDA-approved radioprotective drug to prevent xerostomia following RT. The radioprotective effects of amifostine are thought to be due to its ability to scavenge free radicals [216] (Figure 4). In an open-label phase III clinical trial, Wasserman et al. showed that 2 years post-RT HNC patients who received both RT and amifostine presented with a lower incidence of xerostomia, compared to those receiving RT alone [190]. Additionally, the amifostine group had significantly reduced mouth dryness scores and a significant number of these patients exhibited meaningful unstimulated saliva production. Moreover, amifostine administration with RT did not significantly alter progression-free survival and overall survival rates compared to RT alone [217], a finding supported by a meta-analysis [218]. This is important, given that two major criticisms of amifostine therapy are its toxicity and the possibility that it could reduce the efficacy of RT by protecting cancer cells. In contrast to these promising findings, a randomized double-blind trial reported that amifostine did not affect the incidence of acute or late RT-induced xerostomia (grade ≥ 2) over placebo in HNC patients [219]. A 2017 meta-analysis concluded that there is little evidence that amifostine provides any benefit, and no evidence that reported benefits last longer than 12 months [215]. Additionally, a phase III clinical study by Rades et al. reported that adverse effects of amifostine therapy in combination with RT were responsible for a statistically significant percentage (41%) of patients in the study group discontinuing treatment [220]. The reported clinical benefit of amifostine is questionable and, due to toxicity concerns, amifostine is not widely used [17].

#### 5.3.2. Promising Preclinical Studies

Although there is currently only one FDA-approved radioprotective therapy, there are promising preclinical studies investigating potential radioprotective approaches (Figure 4). We recently reported that antagonism of the ATP-gated ionotropic P2X7 receptor (P2X7R) by i.p. injection of A-438079 in FVB mice provided significant radioprotection and maintained carbachol-induced saliva flow rates similar to non-irradiated mice [48]. P2X7R is highly expressed in mouse salivary glands, where its activation induces pro-inflammatory responses, including membrane blebbing, caspase activation, IL-1β release, recruitment of immune cells and NLRP3 inflammasome assembly [161,225,226]. We also have previously reported that A-438079 attenuates lymphocytic infiltration of SMGs and increases saliva secretion in a mouse model of the autoimmune disease Sjögren’s syndrome [161,227]. Another promising pharmacological approach is the use of tyrosine kinase inhibitors (TKIs). Delivery of TKIs, dasatinib or imatinib, protect the mouse salivary gland from IR-induced damage and loss of function without affecting xenograft tumor growth [78]. This response was due to reduced activation of PKCδ, an important regulator of apoptosis in salivary gland acinar cells [63,77,228,229,230].

Introduction of human neurotrophic factor neurturin (NRTN) using an adeno-associated virus serotype 2 (AAV2) vector prior to IR, but not post-IR, was radioprotective, preventing hyposalivation in both murine and porcine models [54]. NRTN is essential for proper innervation of the salivary glands, which is required for salivary secretory function. RNA sequencing analysis revealed a reduction in expression of fibrotic genes and both innate and humoral immune responses in mice and minipigs receiving AAV2-NRTN [54]. While additional studies are warranted to further investigate immune responses and the duration of improvement of saliva secretion in the IR-treated porcine model, these exciting findings in the highly translational Yucatan minipig model [57,231] are promising for future clinical trials.

### 5.4. Regeneration

There are currently no FDA-approved therapeutics available to patients to restore salivary function after RT. Stem cell therapies to repair or regenerate damaged salivary gland tissue and gene therapy approaches are being studied in preclinical animal models [232]. Additionally, there are two active clinical trials that are testing the efficacy of delivering human aquaporin-1 (hAQP1) to IR-damaged salivary glands to improve secretory function.

#### 5.4.1. Gene Therapy

There are currently two clinical trials investigating the delivery of aquaporin-1 (AQP1) via adeno-associated viral vector 2 (AAV2) to treat IR-induced salivary hypofunction (ClinicalTrials.gov NCT02446249 and NCT04043104). AQP1 is a constitutively active water channel that facilitates secretion of fluid along an osmotic gradient [233]. AQP1 is expressed in the myoepithelial and endothelial cells of the human [234,235,236] and mouse [237] salivary glands and is limited to endothelial cells in the rat SMG [238]. Adenoviral delivery of human AQP1 (AdhAQP1) to rat SMGs by retrograde ductal instillation 3–4 months following IR (17.5 or 21 Gy) resulted in a 2- to 3-fold increase in salivary fluid secretion compared to controls [233]. The minipig closely replicates the structural and functional responses of the human salivary gland to irradiation [57,231] and, thus, is a highly translational model for evaluating novel therapies to prevent or reverse IR-induced salivary gland damage in humans. After determining an 85–90% decrease in saliva flow in minipigs at 16 weeks post-IR (20 Gy), AAV2-hAQP1 was delivered directly to the parotid gland via the Stensen’s duct at 17 weeks post-IR [239]. In contrast to minipigs receiving control vector or saline that continued to exhibit diminished salivary output, minipigs receiving AAV2-hAQP1 had a consistent improvement in saliva secretory volume up to 35% of pre-IR levels by 8 weeks following AAV2-hAQP1 administration. As anticipated, the water channel hAQP1 did not reverse changes in saliva composition induced by IR [239]. Adenoviral delivery of hAQP1 in a phase I clinical trial in patients experiencing RT-induced xerostomia resulted in both short- and long-term improvement of parotid salivary flow and sustained symptomatic relief for 2–3 years [240,241]. In contrast to delivery of exogenous AQP1, forced expression of native AQP1 in human cells, including salivary gland cell lines and primary human salivary progenitor cells, has been achieved by delivery of guide RNAs targeting the promoter region of human AQP1 [242,243].

#### 5.4.2. Stem Cell Therapies

As discussed above, progenitor and/or stem cell populations are essential for regeneration of functional salivary glands in mice [2,55,85,86]. Regeneration of salivary glands using stem cell therapies is a promising approach to ameliorate IR-induced salivary gland dysfunction. Isolation of Sca-1-, c-Kit- and Musashi-1-expressing mouse salivary gland stem cells has been achieved by in vitro culture in 3D salispheres followed by enrichment of stem cells with FACS using c-Kit as a marker [86]. These c-Kit^+^ cells were capable of differentiating into functional amylase-producing acinar cells. This same group then investigated the effect of transplanting salisphere cultures in salivary glands of irradiated (15 Gy) female mice. Ninety days after salisphere transplantation, IR-damaged salivary glands in mice showed similar morphology to non-irradiated glands, with restored acinar cell populations and improved saliva production compared to irradiated, untreated glands [86]. Perhaps most impressive was the number of cells required for restoration, i.e., as few as 300 c-Kit^+^ progenitor cells were capable of restoring salivary gland function [86]. Additional populations of murine salivary stem and/or progenitor cells have been identified that are capable of regenerating salivary gland tissue and rescuing IR-induced hyposalivation in mouse models. As few as 100 CD24^+^c-Kit^+^Sca1^+^ progenitor cells from adult murine SMGs were capable of restoring saliva secretion and functional acini in vivo [244]. Isolated CD24^hi^/Cd29^hi^ adult murine salivary gland progenitor cells were capable of multi-lineage differentiation in vitro and restored salivary function in vivo [245]. While less is known about adult human salivary gland stem cells, a similar c-Kit^+^ stem cell population has been identified [246] that is capable of self-renewal and restoring salivary gland function following irradiation in a murine xenotransplantation model [247]. A number of groups are testing novel biomaterials approaches harnessing the regenerative potential of isolated stem or progenitor cells to engineer implantable tissue [248]. Additionally, one group is using primary murine SMG cells—rather than isolated stem cells—to build cell sheets for salivary gland regeneration [249].

Rather than delivering progenitor cells to IR-damaged glands, other groups are investigating signaling pathways that may restore progenitor cell populations lost in RT (Figure 4). Transient overexpression of Shh restored IR-induced hyposalivation in mice by maintaining salivary stem/progenitor cells [224]. Shh signaling has been shown to be essential for SMG development in mice [250,251] and is activated during regeneration [252]. Activation of the Shh pathway also preserves normal parasympathetic innervation of the SMG [224]. Recently, another group found that depleting senescent salivary gland cells following IR by treatment with the senolytic agent, ABT263, an inhibitor of BCL-2 and BCL-xL that selectively induces apoptosis in senescent cells, at either 8 or 11 weeks post-IR (15 Gy) led to regeneration of aquaporin-5-expressing acinar cells and improved salivary gland function in C57BL/6 mice [68]. Using an in vitro organoid culture model, this group demonstrated that elimination of IR-induced senescent cells enhanced the self-renewal potential of remaining salivary gland stem cells [68]. Another pharmacological approach for preventing IR-induced progenitor cell damage involves administration of insulin-like growth factor 1 (IGF-1). Chibly et al. demonstrated that IGF-1 delivered 4–7 days following irradiation improved saliva production in a PKCζ-dependent manner [55]. Finally, Emmerson et al. identified a SOX2^+^ adult human salivary gland progenitor cell population in all three major salivary glands (submandibular, sublingual and parotid glands) that was capable of differentiating into acinar, but not ductal cells [24]. They demonstrated that SOX2 was essential for salivary gland regeneration following a single 10 Gy dose of γ-radiation to the murine sublingual gland. Using an ex vivo model, SOX2^+^ cells were capable of repopulating the irradiated murine sublingual gland. Furthermore, in human SMG cells, SOX2 expression as well as both acinar and ductal markers were maintained by muscarinic activation [24], suggesting that future studies should target muscarinic signaling as a means to restore residual progenitor cell function after RT.

#### 5.4.3. Pharmacological Approaches

In addition to gene and stem cell therapies, some groups are taking a pharmacological approach to salivary gland regeneration (Figure 4). Rapamycin, an inhibitor of mTOR signaling, is one such agent [88,223]. As discussed earlier, treating FVB mice with the rapamycin analog, CCI-779, on days 4–8 following IR reduced proliferation rates and improved saliva flow rates 30 days post-IR [88]. CCI-779 is an FDA-approved therapy for the treatment of renal cell carcinoma and mantle cell lymphoma [253] and both CCI-779 and rapamycin are currently being investigated in clinical trials for several other cancer types and amyotrophic lateral sclerosis (ALS) (clinicaltrials.gov). Minipigs receiving i.p. injection of rapamycin 1 h prior to RT had improved saliva flow rates 12 weeks post-IR [223], suggesting that targeting mTOR signaling may be beneficial as either a radioprotective or regenerative therapeutic approach. Another potential pharmacological intervention to promote salivary gland regeneration is the post-irradiation delivery of EDAR agonist monoclonal antibodies. EDAR signaling is involved in salivary gland development and transient activation of EDAR signaling post-IR (5 Gy) restores salivary gland function and amylase levels through 90 days in mice [65]. In conclusion, although still in the developmental phase, pharmacological approaches as well as gene and stem cell therapies provide promising new avenues for restoring salivary gland function in HNC patients who have undergone RT.

## 6. Summary

Whether as a result of direct radiation exposure or due to bystander effects, radiotherapy of the head and neck region results in damage to salivary glands that often leads to permanent dysfunction and associated complications, such as increased oral infections, functional impairments in speaking, swallowing, and eating and diminished quality of life. The mechanisms of acute and chronic dysfunction have been investigated using multiple animal models. In the present review, we discussed the available data describing the mechanisms of acute and chronic IR-induced salivary gland dysfunction. In animal models of RT, acute salivary gland dysfunction involves DNA damage and insufficient repair, aberrant calcium signaling, acinar cell apoptosis and ROS production. While these mechanisms contribute to long-term loss of function, sustained salivary dysfunction is further influenced by inflammatory responses, neuronal and vascular changes, loss of epithelial polarity, compensatory proliferation, impaired stem or progenitor cell populations, cytoskeletal rearrangements and fibrosis. One area that deserves further investigation is the contribution of immune cells to IR-induced salivary gland dysfunction. IR-induced immunomodulation is a well-appreciated driver of damage in other models of IR, both with regard to direct IR exposure as well as influencing bystander effects. Further, our overall understanding of bystander effects in the salivary glands following irradiation is inadequate. Building on research performed in other IR-induced damage models, we propose that purinergic signaling through P2 receptors in the salivary gland may be a critical mediator of bystander effects in IR-induced salivary gland dysfunction. At present, artificial saliva and sialagogues provide inadequate and temporary symptom relief in RT. The therapeutic benefit of amifostine, the only FDA-approved radioprotective treatment, is questionable due to toxicity concerns. The need for additional preventative and regenerative approaches is essential. Here, we discussed current and future therapeutic approaches for the treatment or prevention of RT-induced salivary gland damage, including a number of promising radioprotective and regenerative therapies being investigated in preclinical animal models and clinical trials. Radioprotective approaches currently being investigated target many of the signaling pathways discussed in this review, while regenerative approaches include gene therapy, pharmacological interventions and stem cell transplantation.
